# Repetitive transcranial magnetic stimulation combined with cognitive behavioral therapy treatment in alcohol-dependent patients: A randomized, double-blind sham-controlled multicenter clinical trial

**DOI:** 10.3389/fpsyt.2022.935491

**Published:** 2022-10-04

**Authors:** Xiaorui Hu, Tian Zhang, Hongkun Ma, Xuhui Zhou, Hongxuan Wang, Xiaohong Wang, Chang Cheng, Yanfei Li, Ranran Duan, Bo Zhang, Huaizhi Wang, Jia Lu, Chuanyi Kang, Na Zhao, Yingjie Zhang, Lu Tian, Jun Liu, Jingjing Shi, Zhe Wang, Xinxin Zhou, Shuang Zhu, Qingxia Liu, Xuemin Li, Honghui Wang, Mingxuan Nie, Mei Yang, Jianzhong Yang, Yong Chi, Xiaofeng Zhu, Jian Hu, Yanjie Jia, Ying Peng, Lei Liu

**Affiliations:** ^1^Department of Psychiatry, The First Affiliated Hospital of Harbin Medical University, Harbin, Heilongjiang, China; ^2^The First Affiliated Hospital of Zhengzhou University, Zhengzhou, China; ^3^Department of Epidemiology and Health Statistics, Mudanjiang Medical University, Mudanjiang, China; ^4^Hunan Provincial Brain Hospital, Changsha, China; ^5^Sun Yat-sen Memorial Hospital, Sun Yat-sen University, Guangzhou, China; ^6^Beijing Anding Hospital, Capital Medical University, Beijing, China; ^7^The First Psychiatric Hospital of Harbin, Harbin, China; ^8^Shenzhen Mental Health Center, Shenzhen Kangning Hospital, Shenzhen, China; ^9^The Second Affiliated Hospital of Kunming Medical University, Kunming, China; ^10^Department of Physiology and Neurobiology, Mudanjiang Medical University, Mudanjiang, China

**Keywords:** alcohol dependence, repetitive transcranial magnetic stimulation, cognitive behavioral therapy, relapse, combination therapy

## Abstract

**Background:**

Alcohol dependence (AD) is a complex addictive disorder with a high relapse rate. Previous studies have shown that both repetitive transcranial magnetic stimulation (rTMS) and cognitive behavioral therapy (CBT) may be effective for AD, and we aim to explore more effective treatment options to reduce relapse rates for AD.

**Materials and methods:**

A total of 263 AD patients were recruited. They were divided into six groups according to the location and the type of rTMS: left dorsolateral prefrontal cortex (DLPFC), right DLPFC, sham stimulation, and whether they received CBT treatment: with a fixed schedule (C1) and without a fixed plan (C0). There were included in sham rTMS + C0 group (*n* = 50), sham rTMS + C1 group (*n* = 37), right rTMS + C0 group (*n* = 45), right rTMS + C1 group (*n* = 42), left rTMS + C0 group (*n* = 49), left rTMS + C1 group (*n* = 40). We used obsessive compulsive drinking scale (OCDS), visual analogue scale (VAS), alcohol dependence scale (ADS), montreal cognitive assessment (MoCA), generalized anxiety disorder-7 (GAD-7), patient health questionnaire-9 items (PHQ-9), and Pittsburgh sleep quality index (PSQI) to assess alcohol cravings, alcohol dependence, cognition, anxiety, depression, and sleep quality. They were followed up and evaluated for relapse.

**Results:**

The sham rTMS + C0 group relapse rate was significantly higher than the right rTMS + C1 group (*P* = 0.006), the left rTMS + C0 group (*P* = 0.031), the left rTMS + C1 group (*P* = 0.043). The right rTMS + C0 group showed significantly higher relapse rate compared to the right rTMS + C1 group (*P* = 0.046). There was no significant difference in relapse rates between other groups. The repeated-measures ANOVA showed an interaction effect between group and time was significant in the rate of patient health questionnaire-9 items (PHQ-9) scale reduction (*P* = 0.020). Logistic analysis indicated that smoking and alcohol consumption were independent determinants of relapse (*P* < 0.05). At 24 weeks of follow-up, Kaplan–Meier survival analysis reveal that there is statistically significant relapse rate between six groups (*P* = 0.025), left rTMS + C1 group has the best treatment effect for alcohol dependent patients. Cox regression analysis confirmed that current smoking, total cholesterol, and total bilirubin (TBIL) level were risk factors of relapse (*P* < 0.05).

**Conclusion:**

This study is the first to suggest that the combination of rTMS and CBT may be a potentially effective treatment for reducing relapse.

## Introduction

It is currently widely recognized that alcoholism is a complex, multi-dimensional, and multifactorial disease. Notably, 9–17% of drinkers meet the diagnostic criteria for alcohol dependence (AD) (1). AD is a complex addictive disease. In general, addiction formation is closely related to biological factors, and the occurrence and development of addictive behaviors are related to psychological and social factors, such as parenting style (2), family relationships (3), and childhood sexual abuse (4). Because of long-term heavy drinking, the body gradually develops physical and psychological dependence on alcohol, damaging physical, mental, and social functions. AD in all countries in the world is a severe public health problem. With the development of the social economy, and the continuous improvement of people’s living standards, alcohol dependence prevalence is gradually rising. In 2018, the World Health Organization’s global status report on alcohol and health revealed approximately 283 million AD (accounting for 5.1% of the world’s 15 years old and older adults). The prevalence rates vary considerably between countries and are significantly influenced by drinking culture and social norms. Europe has the highest prevalence (8.8% of the adult population), followed by the United States (8.2% of the adult population), while the prevalence of alcohol dependence in China is 2.3% (5). AD is among the highest risk factors for shortening the life cycle and leads to more than 200 health conditions, with a high disease burden, high disability rate, and high mortality (6).

Early long-term AD treatment mainly included drug and behavioral therapy (7). However, these treatments are only moderately helpful, and more than 50% of treated patients relapse within 1 year (8). Even though research over the past 50 years has demonstrated that addiction is a brain disease, we still have no effective treatments based on neural circuits and specific neural targets that directly and specifically target AD. For the first time, a non-invasive neuroendocrine technique called transcranial magnetic stimulation (TMS) appears to be the primary physical therapy approach to fill this gap in developing AD treatment. Dr. Anthony Barker invented TMS technology in Sheffield, UK, in 1984 (9). TMS is based on electromagnetic induction, a brief focused electromagnetic pulse penetrating the skull without attenuation and stimulating the targeted brain region. Typically, the magnetic field is high enough to induce depolarization of neurons in the cortex region where the coils are located. This technique is called repetitive TMS (rTMS) when TMS pulses are transmitted continuously and repeatedly at a precise frequency. High-frequency repeat transcranial magnetic stimulation (HF-rTMS) (>5 Hz) promoted, while low frequency rTMS (<1 Hz) inhibited motor cortex excitability (10). rTMS can induce changes in brain function for a more extended period by modulating cortical excitability, neurotransmitters, and neuronal plasticity, reducing the desire for addictive substances, improving cognitive function, and ultimately reducing the relapse rate (11). It provides a new therapeutic advantage for alcohol dependence unmatched by pharmacotherapy.

Brodmann regions 9 and 46 are often known as the dorsolateral prefrontal cortex (DLPFC). DLPFC plays an essential role in decision making, reasoning, working memory, inhibition, and outcome prediction, plays a vital role in substance-related cravings, including alcohol, and has gained a recognized reputation as a successful treatment target for TMS in patients with depression (12). Previous studies have shown that rTMS, DLPFC was selected as the stimulation site to show promise for the reduction of craving and substance use in nicotine (13) and cocaine addiction (14), as well as in behavioral addictions (15), which are showing significant development potential. All these suggest that DLPFC may be an ideal target for TMS treatment of AD. However, the treatment duration, frequency of stimulation, and intensity of stimulation frequency of rTMS have not been determined. Also, it has not been clearly answered whether left, right, or bilateral stimulation is the most effective method (16–20).

Another critical aspect of TMS treatment for AD is combining it with existing behavioral therapies for AD. Ultimately, all treatments for AD need to emphasize changing behavior. Cognitive behavioral therapy (CBT) has become a front-line behavioral therapy for AD and other substance use disorders (SUDs) in recent years (21). Drawn up in the 1960s and 1970s in the United States, CBT is a time-limited, multistage intervention designed to address the cognitive, emotional, and environmental risks of drug use, identify and change unreasonable beliefs, and provide training in behavioral self-control skills. By overcoming the desire to seek out and consume alcohol and by dealing with situations that might trigger these desires, to improve patients’ psychological defense ability and build psychological defense mechanisms to help individuals achieve and maintain abstinence or reduce drinking cravings (22). A meta-analysis found that Internet-based alcohol interventions guided by health professionals were more effective than unguided (fully automated) interventions (23). However, therapist-guided interventions were not more effective than self-help interventions in the two most recent studies on internet-based cognitive behavioral therapy for alcohol (24, 25). Therefore, the trial hypothesizes that a therapist-guided, fixed-plan model CBT would be more effective in reducing relapse rates than simply providing a standardized basic interview of about 10 min without any therapeutic intervention.

A large part of the disease burden is due to the ongoing effects of alcohol on the central nervous system (26). As a central nervous system depressant, alcohol can, directly and indirectly, act on the central nervous system, leading to cognitive dysfunction (27). Cognitive impairment has increasingly become the focus of AD research. Depending on studies, 50–80% of AD patients have cognitive impairment (28). In addition, people with AD often experience an intense, uncontrollable desire for alcohol, also known as craving (29). Combining perceived desires with reduced cognitive control can lead to problems managing cravings, leading to relapse (30). AD is often associated with various psychiatric and social behavioral comorbidities, including severe sleep disorders, anxiety, and depression (31, 32).

Therefore, the biggest problem plaguing AD is its extraordinary relapse rate. There are limited treatment methods for AD relapse, and a high relapse rate will hinder the treatment effect. Depending on statistics, although the early treatment is beneficial, up to 85% of AD patients still drink again (33), significantly the highest within 6–12 months after treatment (34). This suggests a critical need to understand factors associated with relapse. The evidence is inconsistent regarding the potential impact of smoking on recovery from alcohol dependence. Although some studies have found that smoking negatively affects the treatment outcome of alcohol dependent patients (35), however, there is also evidence that smoking does not pose a risk to sobriety in AD (36). Variables associated with nicotine dependence may be predictors of future alcohol dependence (37). Alcohol biomarkers have become valuable tools for objectively assessing treatment outcomes (38). Routine blood tests may help predict the long-term development of alcohol withdrawal treatment and may become a more feasible and cost-effective method for assessing relapse risk (39). This study explored the relationship between pre-treatment predictors (demographics and laboratory tests), post-treatment predictors (rTMS and CBT), and relapse. We hypothesized that treatment-related variables would be best helpful in predicting the prognosis of alcohol dependent patients receiving treatment. Even though AD profoundly impacts individuals’ work, social life, and interpersonal relationships (40), the treatment rate of AD is extremely low, and the establishment of effective treatment is essential.

Although rTMS and CBT have positive effects on all dimensions of AD, there is currently more heterogeneity in the outcomes of rTMS and CBT for AD compared with pharmacological AD treatment. There are no studies on whether there is an advantage of combined treatment with CBT and rTMS. Therefore, we conducted a randomized, double-blind sham-controlled multicenter clinical trial in which sham rTMS, left DLPFC rTMS, right DLPFC rTMS, and combined with CBT were utilized to treat AD patients. The ADS, VAS, OCDS, PHQ-9, GAD-7, PSQI, and MoCA scales were regularly used to evaluate the patients’ alcohol dependence, drinking desire, cognitive function anxiety, depression, and sleep. And during the follow-up period, whether the patients relapsed were recorded by a self-assessment diary of alcohol consumption. The primary objectives of this study: assess the effectiveness of rTMS in combination with CBT for AD in reducing relapse and investigate the risk factors of relapse. The secondary objectives of this study: whether different treatment modalities improve anxiety, depression, cognitive function, and craving and indirectly reduce the rate of relapse to drinking, and provide new directions for AD treatment.

## Materials and methods

### Participants

We selected patients with AD who were outpatients and inpatients at the First Affiliated Hospital of Harbin Medical University, Sun Yat-sen Memorial Hospital, the First Affiliated Hospital of Zhengzhou University, Mudanjiang Medical University, Beijing Anding Hospital Affiliated to Capital Medical University, Shenzhen Kangning Hospital, Hunan Provincial Brain Hospital, the Second Affiliated Hospital of Kunming Medical University from March 2019 to September 2021 as the study population. The Ethics Committee endorsed the study, and all participants obtained informed consent from themselves and signed informed consent. Supplementary Figure 1 shows the CONSORT diagram of the flow of participants through the trial.

Inclusion criteria: (1) 18–65 years old; (2) Meet the diagnostic criteria of DSM-IV alcohol dependence; (3) No history of neurological diseases or family history of mental disorders.

Exclusion criteria: (1) Clinical Institute Alcohol Withdrawal Syndrome Scale (CIWA-Ar) > 9 points in acute alcohol withdrawal reaction stage; (2) Severe neurological or mental diseases caused by other diseases other than chronic alcohol dependence, such as stroke, intracranial infection, brain tumors, schizophrenia, etc.; (3) Have experienced a traumatic brain injury or other brain tissue damage; (4) Is taking or has taken any other psychotropic drugs or is dependent on other drugs or other substances; (5) Contraindications of rTMS therapy: a. Acute infectious diseases; b. Presence of metallic foreign bodies in the skull; c. After craniotomy; d. Intracranial aneurysm or other vascular malformation; e. Epilepsy history; f. Severe cardiovascular disease, especially those with pacemakers or cardiac stents.

Experimental termination criteria: (1) Severe adverse reactions occurred during the study; (2) Subjects did not cooperate with treatment and had poor compliance.

### Research methods

#### General clinical data

We collected general clinical data: self-designed case report form, general physical examination, basic vital signs, hematology routine, and blood biochemistry test results. And during the follow-up period, whether the patients relapsed were recorded by a self-assessment diary of alcohol consumption. Based on the self-assessment diary of alcohol consumption, combined with regular telephone follow-up with family members at week 0, week 2, week 8, week 12, and week 24, and outpatient follow-up, to ensure the authenticity of the self-assessment diary of alcohol consumption.

(1) Self-designed case report form, including gender, age, drinking years, alcohol consumption, drinking type, frequency of alcohol consumption, and current smoking.
(2) General physical examination and basic vital signs, including body mass index, heart rate, systolic blood pressure, and diastolic blood pressure.
(3) Hematology routine including white blood cell, red-blood-cell, Platelets, and hemoglobin.
(4) Blood biochemistry, including fasting glucose, uric acid, serum creatinine, alanine transaminase, aspartate aminotransferase, gamma-glutamyl transpeptidase, total bilirubin, direct bilirubin, indirect bilirubin, Total cholesterol, Triglycerides, low-density lipoprotein, high-density lipoprotein.

####  Treatment plan

(1) Repetitive transcranial magnetic stimulation procedure:

Treatment device: Use of “8” coil transcranial magnetic stimulation instrument (using the YiRuiDe^®^ CCY-1 classic magnetic stimulator device; YiRuiDe Group, Wuhan, China).

Treatment duration: Starting at baseline, stimulated on five consecutive days (W1), suspended on weekends, treatment continued for five consecutive working days in the second week (W2), total of 10 sessions.

Treatment: The individual motor threshold (MT) for the right/left abductor pollicis brevis muscle was determined using single-pulse TMS in combination with motor-evoked potentials (MEP). The MT was considered the lowest intensity to induce a visual MEP on electromyography (EMG). A stimulation intensity of 110% of the subject’s resting MT was used for the study. a. Left DLPFC rTMS: the Left dorsolateral prefrontal cortex was selected as the stimulation site, and the location was determined by the international 10–20 electroencephalography system (the location of the left DLPFC corresponds to the F3). Treatment parameters were set (stimulation intensity: 110% threshold, stimulation frequency: high-frequency 10 Hz, train duration: 5 s, intertrain interval: 20 s, total trains per session: 30 trains, total 10 sessions). b. Right DLPFC rTMS: the Right dorsolateral prefrontal cortex was selected as the stimulation site. The international 10–20 electroencephalogram system was used for localization (the location of the right DLPFC corresponds to the F4). The treatment parameters were the same as the left rTMS group. c. Sham rTMS: The spiral edge was placed at the stimulation site, and the stimulation intensity was set to 0 or 1%. The other treatment parameters were fixed as above. All subjects were unaware of the type of stimulation they received; they wore earplugs. The study was conducted in conformity with the current safety guidelines (41).

(2) Cognitive behavioral therapy procedure:

Treatment duration: Starting at baseline, once a week for 8 weeks (W1–W8), for a total of eight sessions per subject.

Treatment: Cognitive behavioral therapy with a fixed plan is 60 min per session, and each session is divided into three phases preparation, work, and summary, each phase being 20 min. According to the abstinence treatment research paradigm (pre-action stage, planning stage, preparation stage, action stage, maintenance/consolidation stage, and termination/relapse stage), this study designed eight individual cognitive-behavioral therapy sessions with different themes. The eight themes were: a. Individual motivational feedback; b. Identification and handling of predisposing factors; c. Transformation of negative cognition; d. Negative emotion control and management; e. Enrichment of drink-refusal skills; f. Improvement of interpersonal relationships; g. Establishment of a recovery support system; h. Reduction of relapse risk. The CBT group, without a fixed plan, provided only about 10 min of a standardized basic interview without any therapeutic intervention. The CBT treatment protocol was designed with reference to the studies of Johansson M (42) and Magill M (43), with appropriate adaptations. One of the treatment regimens was randomly assigned to CBT with a fixed plan (C1); CBT without a fixed plan (C0).

(3) Treatment as usual:

All subjects received routine drug therapy with the same treatment period, and dose, including the use of mecobalamin and vitamin B to nourish nerves, antioxidant damage with vitamin C and vitamin E. Temporary short-term low-dose Benzodiazepines were given to patients when necessary.

#### Research group

(1) Randomization and double-blind method: a clinical research assistant who is not involved in other clinical treatment, scale, and outcome evaluation will automatically randomize subjects by the computer algorithm. Neither the subject nor the clinical investigator knew which treatment group the subject was assigned. TMS investigators, and CBT study personnel, are unaware of changes in subject outcomes.

(2) This study was divided into six groups:

a. TAU + sham rTMS + CBT without a fixed plan (sham rTMS + C0 group).
b. TAU + sham rTMS + CBT with a fixed plan (sham rTMS + C1 group).
c. TAU + right DLPFC rTMS + CBT without a fixed plan (right rTMS + C0 group).
d. TAU + right DLPFC rTMS + CBT with a fixed plan (right rTMS + C1 group).
e. TAU + left DLPFC rTMS + CBT without a fixed plan (left rTMS + C0 group).
f. TAU + left DLPFC rTMS + CBT with a fixed plan (left rTMS + C1 group).

#### Scale evaluation

The CIWA-Ar (44) assessment was performed at enrollment to exclude patients in the acute alcohol withdrawal phase. The obsessive-compulsive drinking scale (OCDS) (45) and visual analogue scale (VAS) (46) were administered to measure the severity of alcohol cravings. The alcohol dependence scale (ADS) (47) was used to assess the severity of alcohol dependence. Montreal Cognitive Assessment (MoCA) (48) was used to measure the overall cognitive level of the patients. Pittsburgh Sleep Quality Index (PSQI) (49) was utilized to evaluate the Sleep Quality of patients. Generalized anxiety disorder-7 (GAD-7) (50) was used to assess the severity of anxiety symptoms. Patient Health Questionnaire-9 items (PHQ-9) (51) assess the patient’s depressive symptoms. The above scales of OCDS, VAS, PSQI, GAD-7, PHQ-9 were evaluated at week 0, week 2, week 8, week 12, and week 24. MoCA was evaluated at week 0, week 8, week 12, and week 24. ADS was evaluated at week 0, week 12, and week 24.

#### Statistical analysis

The Kolmogorov–Smirnov test was used to test normal distribution. Continuous variables were expressed as the mean ± standard deviation. One-way analysis of variance (ANOVA) model was used to analyze the differences in the treatment effects of different treatment regimens on relapse in AD patients. Categorical variables were analyzed using the chi-squared test or Fisher’s exact test. Repeated measures ANOVA was used to explore the interaction of different treatment modalities with changes in the rate of scale score reduction over treatment time, using baseline period, 2 weeks, 2 months, 3 months, and 6 months of treatment scores as relevant measures. And further simple effect analysis was performed for a significant interaction effect between group and time. Binary logistic regression analysis was performed to identify independent variables influencing relapse. Relapse rate of six groups were compared using Kaplan–Meier survival analysis. Risk factors for the relapse rate were assessed using Cox regression model analysis. Statistical analyses were performed in SPSS-23 (IBM). Insert additional missing values using the maximum expected value method. Differences between the groups were considered statistically significant at *P* < 0.05.

## Results

### Clinical characteristics

A total of 297 subjects were included in this study, of which 34 were excluded according to the experimental termination criteria, and 263 subjects were finally included in the analysis. There were included in sham rTMS + C0 group (*n* = 50), sham rTMS + C1 group (*n* = 37), right rTMS + C0 group (*n* = 45), right rTMS + C1 group (*n* = 42), left rTMS + C0 group (*n* = 49), left rTMS + C1 group (*n* = 40). Table 1 shows the comparison of the baseline data between the six groups. Regarding demographic characteristics, laboratory tests scale, and assessment at baseline, there was no statistically significant difference.

**TABLE 1:** Comparison of basic clinical information and laboratory test levels of patients recruited for the study.

	Sham rTMS + C0	Sham rTMS + C1	Right rTMS + C0	Right rTMS + C1	Left rTMS + C0	Left rTMS + C1	*P*
**Demographics**							
Age, years	48 ± 10	46 ± 10	47 ± 12	44 ± 10	45 ± 11	48 ± 11	0.409
Male, n%	34 (87.2)	28 (96.6)	29 (85.3)	32 (97)	36 (94.7)	28 (84.8)	0.253
Drinking duration (year)	22 ± 10	23 ± 11	23 ± 11	21 ± 10	20 ± 11	24 ± 12	0.627
Frequency of drinking (day/week)	5.5 ± 2.1	5.2 ± 2.2	5.8 ± 1.8	5.6 ± 2.0	5.8 ± 1.8	5.9 ± 1.7	0.647
Alcohol consumption, ml	930 ± 1028	756 ± 1164	1141 ± 1327	983 ± 1380	894 ± 964	849 ± 873	0.735
Current smoking	39 (78)	27 (73)	26 (57.8)	29 (69)	28 (57.1)	23 (57.5)	0.130
BMI, kg/m^2^	23.12 ± 3.37	23.23 ± 3.37	22.80 ± 3.74	23.54 ± 3.62	22.52 ± 4.79	22.20 ± 3.59	0.625
Heart rate, bpm	87.77 ± 14.69	87.89 ± 12.55	90.44 ± 12.61	86.96 ± 16.13	90.38 ± 17.30	84.67 ± 15.72	0.471
Systolic blood pressure, mmHg	128.46 ± 13.33	133.78 ± 21.50	131.54 ± 14.05	130.82 ± 16.14	133.64 ± 18.43	130.10 ± 17.52	0.636
Diastolic blood pressure, mmHg	83.98 ± 10.60	85.32 ± 12.28	84.27 ± 9.46	84.01 ± 12.46	86.75 ± 11.22	85.22 ± 9.45	0.811
**Laboratory tests**							
WBC, ×10^9^/L	7.05 ± 1.956	7.18 ± 2.776	6.60 ± 1.882	6.27 ± 1.879	6.93 ± 1.779	6.54 ± 1.660	0.272
RBC, ×10^12^/L	4.57 ± 0.84	4.63 ± 0.676	4.30 ± 0.543	4.47 ± 0.557	4.35 ± 0.610	4.31 ± 0.465	0.068
Platelets, ×10^9^/L	229.41 ± 58.785	227.52 ± 70.213	230.57 ± 72.348	192.70 ± 66.912	230.82 ± 79.573	221.07 ± 96.455	0.137
HGB, g/L	141.97 ± 25.480	144.10 ± 21.102	139.87 ± 16.004	141.33 ± 15.767	141.32 ± 17.014	140.35 ± 18.670	0.949
Fasting glucose, mmol/L	4.42 ± 9.539	12.29 ± 50.617	5.06 ± 7.823	5.99 ± 6.323	5.86 ± 10.818	7.73 ± 16.886	0.614
UA, μmol/L	439.97 ± 760.166	312.80 ± 269.687	373.30 ± 168.878	315.02 ± 175.239	363.24 ± 212.268	317.07 ± 177.631	0.556
Serum creatinine, μmol/L	65.32 ± 12.976	64.93 ± 12.900	66 ± 16.081	66.81 ± 13.458	71.92 ± 14.460	68.41 ± 13.625	0.155
ALT, U/L	30.74 ± 27.301	27.21 ± 16.937	40.87 ± 39.751	43.36 ± 61.099	45 ± 41.402	40.99 ± 37.858	0.217
AST, U/L	40.12 ± 44.113	35.39 ± 38.326	49.43 ± 57.091	50.99 ± 57.294	53.98 ± 61.308	49.94 ± 48.022	0.541
GGT, U/L	166.08 ± 274.690	90.66 ± 125.281	133.48 ± 201.295	146.97 ± 214.172	131.05 ± 284.344	103.28 ± 128.302	0.643
TBIL, μmol/L	17.83 ± 10.153	14.65 ± 6.789	16.46 ± 18.047	19.27 ± 16.250	15.25 ± 11.226	13.00 ± 10.756	0.264
DBIL, μmol/L	5.40 ± 3.675	5.67 ± 5.003	4.96 ± 4.721	6.64 ± 6.858	4.62 ± 3.939	4.91 ± 3.853	0.411
IBIL, μmol/L	12.35 ± 7.725	9.58 ± 5.25	11.45 ± 15.212	11.62 ± 9.116	10.55 ± 8.830	8.72 ± 6.648	0.488
Total cholesterol, mmol/L	15.90 ± 65.423	8.81 ± 12.813	3.50 ± 14.260	5.73 ± 12.382	4.72 ± 9.807	6.28 ± 12.745	0.397
Triglycerides, mmol/L	1.91 ± 1.160	1.90 ± 1.391	1.87 ± 1.717	1.64 ± 1.049	1.81 ± 1.153	1.70 ± 0.859	0.896
LDL, mmol/L	2.61 ± 0.874	2.82 ± 0.888	2.77 ± 0.957	2.51 ± 0.820	2.56 ± 1.111	2.55 ± 0.869	0.566
HDL, mmol/L	1.42 ± 0.616	1.21 ± 0.630	1.41 ± 0.577	1.37 ± 0.458	1.41 ± 0.624	1.33 ± 0.471	0.562
**Scale evaluation**							
MoCA	17.442 ± 9.028	19.679 ± 8.319	18.730 ± 8.251	19.019 ± 9.055	20.015 ± 8.981	19.610 ± 9.428	0.756
ADS	14.781 ± 9.189	15.742 ± 9.680	17.515 ± 9.120	14.246 ± 9.676	14.863 ± 9.659	14.748 ± 10.542	0.648
CIWA-Ar	9.407 ± 6.889	10.108 ± 10.452	9.056 ± 7.654	8.926 ± 8.640	10.494 ± 8.240	6.566 ± 6.547	0.306
GAD-7	5.14 ± 4.540	4.744 ± 4.487	4.240 ± 3.717	5.463 ± 5.611	5.736 ± 4.290	5.517 ± 4.557	0.645
PHQ-9	7.049 ± 6.054	7.796 ± 6.129	6.577 ± 5.830	7.512 ± 7.694	7.664 ± 6.149	6.253 ± 5.763	0.841
PSQI	9.18 ± 7.870	10.379 ± 8.773	9.609 ± 7.708	8.997 ± 9.662	10.743 ± 11.005	9.431 ± 9.229	0.935
VAS	4.103 ± 3.429	4.732 ± 3.550	3.939 ± 3.483	3.887 ± 3.245	3.839 ± 3.513	4.178 ± 3.144	0.869
OCDS	18.470 ± 10.546	17.731 ± 9.535	18.924 ± 8.685	17.454 ± 11.290	18.880 ± 9.018	18.923 ± 11.084	0.971

C1, cognitive behavioral therapy with a fixed schedule; C0, cognitive behavioral therapy without a fixed plan; BMI, body mass index; WBC, white blood cell; RBC, red-blood-cell; HGB, hemoglobin; UA, uric acid; ALT, alanine transaminase; AST, aspartate aminotransferase; GGT, gamma-glutamyl transpeptidase; TBIL, total bilirubin; DBIL, direct bilirubin; IBIL, indirect bilirubin; HDL, high-density lipoprotein; LDL, low-density lipoprotein; MoCA, Montreal Cognitive Assessment Scale; ADS, Alcohol dependence scale; CIWA-Ar, Clinical Institute Alcohol Withdrawal Syndrome Scale; GAD-7, Generalized anxiety disorder-7; PHQ-9, Patient Health Questionnaire-9 items; VAS, Visual Analogue Scale; OCDS, Obsessive Compulsive Drinking Scale; PSQI, Pittsburgh Sleep Quality Index.

### One-way ANOVA models for relapse rate between the six groups

The sham rTMS + C0 group relapse rate was significantly higher than the right rTMS + C1 group (*P* = 0.006), the left rTMS + C0 group (*P* = 0.031), the left rTMS + C1 group (*P* = 0.043); the right rTMS + C0 group relapse rate was significantly higher than the right rTMS + C1 group (*P* = 0.046) (Table 2). There was no significant difference in relapse rates between other groups.

**TABLE 2:** One-way ANOVA models for relapse rate between the six groups.

Groups	Mean ± SD	Groups	Mean ± SD	*P*-value
Sham rTMS + C0	0.450 ± 0.5038	Right rTMS + C1	0.143 ± 0.3563	0.006
		Left rTMS + C0	0.219 ± 0.4200	0.031
		Left rTMS + C1	0.222 ± 0.4237	0.043
Right rTMS + C0	0.371 ± 0.4902	Right rTMS + C1	0.143 ± 0.3563	0.046

C1, cognitive behavioral therapy with a fixed schedule; C0, cognitive behavioral therapy without a fixed plan.

### Repeated measures ANOVA for the scale score reduction rate in the six groups of patients after 2 weeks, 2 months, 3 months, and 6 months of follow-up

For the reduction rates of GAD, MoCA, OCDS, and PSQI scores among the six groups of alcohol dependent patients, repeated measures ANOVA showed no main effect on the group but a significant main effect of time (Table 3). Also, there was a significant interaction effect between group and time in the rate of PHQ-9 scale score reduction rate (*F* = 3.001, *P* = 0.020), and both the main effect of group and the main effect of time were significant (*F* = 2.492, *P* = 0.032; *F* = 2.918, *P* = 0.037). Further simple effect analysis revealed that, at week 2 of follow-up, the right rTMS + C0 group had a significantly higher PHQ-9 scale score reduction rate than the sham rTMS + C1 group (*P* = 0.039); at week 2 of follow-up, the left rTMS + C0 group had a significantly higher PHQ-9 scale score reduction rate than the right rTMS + C0 group (*P* = 0.011); the right rTMS + C0 group had a significantly higher PHQ-9 scale score reduction rate at week 12 of follow-up than week 2 of follow-up (*P* = 0.046).

**TABLE 3:** Repeated measures ANOVA for the rate of scale score reduction in the six groups of patients after 2 weeks, 2 months, 3 months, and 6 months of follow-up.

Scales	Groups	2 weeks	2 months	3 months	6 months	Group main effect	Time main effect	Time × Group interaction effect
ADS	Sham rTMS + C0			0.192 ± 0.530	0.321 ± 0.991	*F* = 0.857, *P* = 0.511	*F* = 0.339, *P* = 0.799	*F* = 1.276, *P* = 0.279
	Sham rTMS + C1			0.055 ± 1.096	0.045 ± 0.434			
	Right rTMS + C0			0.030 ± 0.520	0.364 ± 0.780			
	right rTMS + C1			0.083 ± 0.527	−0.198 ± 1.873			
	Left rTMS + C0			0.077 ± 0.354	0.153 ± 0.365			
	Left rTMS + C1			0.139 ± 0.357	0.009 ± 0.511			
GAD-7	Sham rTMS + C0	0.129 ± 0.807	0.150 ± 0.366	0.034 ± 0.994	0.124 ± 0.416	*F* = 1.046, *P* = 0.391	*F* = 3.954, *P* = 0.047	*F* = 1.185, *P* = 0.377
	Sham rTMS + C1	0.057 ± 0.667	0.136 ± 0.475	−0.008 ± 0.520	0.166 ± 0.490			
	Right rTMS + C0	0.215 ± 1.440	−0.048 ± 0.895	0.136 ± 0.556	0.042 ± 0.647			
	Right rTMS + C1	0.028 ± 0.833	−0.0783 ± 1.379	0.159 ± 0.436	0.005 ± 0.893			
	Left rTMS + C0	0.048 ± 0.565	0.144 ± 0.683	0.047 ± 0.595	0.123 ± 0.591			
	Left rTMS + C1	0.036 ± 0.919	0.016 ± 1.364	0.177 ± 0.384	0.110 ± 0.604			
MoCA	Sham rTMS + C0		−1.693 ± 5.168	0.038 ± 0.439	−0.115 ± 0.559	*F* = 0.906, *P* = 0.477	*F* = 8.533, *P* = 0.001	*F* = 1.477, *P* = 0.126
	Sham rTMS + C1		−0.810 ± 2.262	−0.184 ± 0.994	−0.032 ± 0.182			
	Right rTMS + C0		−0.861 ± 2.696	−0.023 ± 0.241	−0.054 ± 0.345			
	Right rTMS + C1		−0.079 ± 2.467	−0.028 ± 0.174	−0.013 ± 0.161			
	Left rTMS + C0		−0.505 ± 2.042	−0.020 ± 0.083	−0.014 ± 0.130			
	Left rTMS + C1		−0.889 ± 3.690	−0.215 ± 1.006	−0.050 ± 0.147			
OCDS	Sham rTMS + C0	0.008 ± 0.608	0.117 ± 0.846	−0.006 ± 0.780	0.118 ± 0.820	*F* = 0.345, *P* = 0.885	*F* = 3.543, *P* = 0.005	*F* = 1.199, *P* = 0.386
	Sham rTMS + C1	0.006 ± 0.865	0.255 ± 0.385	0.078 ± 0.448	−0.087 ± 1.111			
	Right rTMS + C0	0.080 ± 0.408	0.151 ± 0.425	−0.010 ± 0.568	−0.172 ± 0.822			
	Right rTMS + C1	0.163 ± 0.394	0.187 ± 0.388	0.132 ± 0.490	−0.085 ± 1.080			
	Left rTMS + C0	0.159 ± 0.419	0.204 ± 0.436	0.138 ± 0.317	−0.088 ± 0.737			
	Left rTMS + C1	0.047 ± 0.634	0.184 ± 0.413	−0.141 ± 1.986	0.037 ± 0.519			
PHQ-9	Sham rTMS + C0	0.008 ± 0.678	0.077 ± 0.698	0.226 ± 0.514	0.079 ± 0.504	*F* = 2.492, *P* = 0.032	*F* = 2.918, *P* = 0.037	*F* = 3.001, *P* = 0.020
	Sham rTMS + C1	0.078 ± 0.457	0.209 ± 0.337	0.113 ± 0.421	0.060 ± 0.697			
	Right rTMS + C0	0.135 ± 1.263	−0.094 ± 1.141	0.182 ± 0.428	−0.014 ± 0.757			
	Right rTMS + C1	0.137 ± 0.385	0.103 ± 0.713	0.178 ± 0.492	−0.047 ± 0.623			
	Left rTMS + C0	0.251 ± 0.359	0.185 ± 0.507	0.123 ± 0.495	−0.020 ± 0.508			
	Left rTMS + C1	0.028 ± 0.701	0.087 ± 0.761	0.138 ± 0.216	0.124 ± 0.408			
PSQI	Sham rTMS + C0	0.151 ± 0.884	0.008 ± 0.605	0.098 ± 0.951	−0.060 ± 0.620	*F* = 0.788, *P* = 0.559	*F* = 2.299, *P* = 0.004	*F* = 0.731, *P* = 0.256
	Sham rTMS + C1	0.500 ± 2.207	0.188 ± 0.424	0.101 ± 0.367	0.004 ± 0.374			
	Right rTMS + C0	0.059 ± 0.551	0.006 ± 0.521	0.047 ± 0.373	0.003 ± 0.537			
	Right rTMS + C1	0.184 ± 1.068	0.142 ± 0.350	0.071 ± 0.457	0.090 ± 0.468			
	Left rTMS + C0	0.036 ± 0.359	0.205 ± 0.484	0.045 ± 0.299	−0.106 ± 0.813			
	Left rTMS + C1	0.167 ± 1.144	0.103 ± 0.514	−0.014 ± 0.445	0.019 ± 0.410			
VAS	Sham rTMS + C0	0.112 ± 1.380	0.222 ± 0.407	0.216 ± 0.414	0.092 ± 0.516	*F* = 0.243, *P* = 0.943	*F* = 1.406, *P* = 0.161	*F* = 1.864, *P* = 0.154
	Sham rTMS + C1	0.133 ± 0.447	0.226 ± 0.278	0.193 ± 0.365	0.209 ± 0.686			
	Right rTMS + C0	0.042 ± 0.585	0.202 ± 0.434	0.176 ± 0.326	0.087 ± 0.477			
	Right rTMS + C1	0.193 ± 0.552	0.220 ± 0.591	0.148 ± 0.352	0.090 ± 0.518			
	Left rTMS + C0	0.194 ± 0.430	0.221 ± 0.892	0.044 ± 0.448	0.094 ± 0.589			
	Left rTMS + C1	0.085 ± 0.412	0.156 ± 0.383	0.148 ± 0.489	0.091 ± 0.593			

C1, cognitive behavioral therapy with a fixed schedule; C0, cognitive behavioral therapy without a fixed plan; MoCA, Montreal Cognitive Assessment Scale; ADS, Alcohol dependence scale; GAD-7, Generalized anxiety disorder-7; PHQ-9, Patient Health Questionnaire-9 items; VAS, Visual Analogue Scale; OCDS, Obsessive Compulsive Drinking Scale; PSQI, Pittsburgh Sleep Quality Index.

### Correlation and Binary logistic regression models for relapse

Relapse was positively correlated with alcohol consumption (*r* = 0.186, *P* = 0.011), white blood cell (*r* = 0.182, *P* = 0.013), hemoglobin (*r* = 0.176, *P* = 0.017), current smoking (*r* = 0.170, *P* = 0.021), CBT (*r* = −0.169, *P* = 0.022). Binary logistic analysis indicated that current smoking (*P* = 0.038) and alcohol consumption (*P* = 0.009) was independent determinant of relapse (Table 4).

**TABLE 4:** Statistically significant results in Binary logistic regression models for relapse.

	B	S.E.	Sig.	OR	95% CI	
					Lower	Upper
Current smoking	−1.329	0.642	0.038	0.265	0.075	0.931
Alcohol consumption, ml	0.000360	0.000150	0.009	1.000	1.000	1.001
CBT	0.528	0.456	0.246	1.696	0.694	4.143
Constant	−0.075	1.649	0.964	0.927		

CBT, cognitive behavioral therapy; CI, confidence ration; OR, odds ratio; SE, standard error.

### Total cholesterol, total bilirubin level, and current smoking were risk factors for relapse

At 24 weeks of follow-up, Kaplan−Meier survival analysis reveals a statistically significant relapse rate between six groups (*P* = 0.025), left rTMS + C1 group has the best treatment effect for alcohol dependent patients (Supplementary Figure 2). Cox regression analysis showed that current smoking (β = 0.835, hazard ratio = 2.306, *P* = 0.045), total cholesterol (β = 0.006, hazard ratio = 1.006, *P* = 0.034), and TBIL (β = 0.025, hazard ratio = 1.025, *P* = 0.026) level were risk factors of relapse (Table 5).

**TABLE 5:** Statistically significant results in Cox regression analyses for predictors of relapse.

Variable	Univariate analysis		Multivariate analysis	
	HR (95% CI)	*P*-value	HR (95% CI)	*P*-value
WBC	1.133	0.033	1.077	0.267
RBC	1.977	0.006	1.260	0.462
HGB	1.019	0.010	1.010	0.306
TBIL	1.034	0.000	1.025	0.026
Total cholesterol	1.006	0.007	1.006	0.034
Current smoking	2.805	0.011	2.306	0.045

WBC, white blood cell; RBC, red-blood-cell; HGB, hemoglobin; TBIL, total bilirubin; CI, confidence interval; HR, hazard ratio.

## Discussion

Most importantly, let’s talk about the combination of rTMS and CBT. The effectiveness of CBT for AD has been demonstrated in an extensive review of psychosocial therapies (52, 53). The number of days of heavy drinking dropped significantly after CBT, according to new research (54). The combination of rTMS and CBT has been shown to be more effective than treatment strategies alone in patients with major depression (55), and a shorter course of treatment can achieve remission (56). To date, no studies have investigated the efficacy of this promising approach in AD. Our results are the first to show that rTMS combined with CBT is superior to rTMS alone in reducing the rate of relapse. At 24 weeks of follow-up, Kaplan−Meier survival analysis reveals a statistically significant relapse rate between six groups, left rTMS + C1 group has the best treatment effect for alcohol dependent patients. CBT has been proposed to function at the neural level through DLPFC (57). Therefore, stimulation of left DLPFC by rTMS may synergistically enhance the effect of CBT, inducing neural plasticity and making neural circuits recover faster (58). It is also hypothesized that CBT may provide a “foundation” for treatment to improve treatment retention and adherence and address other ancillary issues. So it makes more sense to combine these two approaches, making it possible to have more powerful stacking effects and more prolonged-lasting effects.

Anxiety and depression of AD patients may be aggravated due to decreased self-control, poor social support system, and deteriorating quality of life. At the same time, AD patients will show withdrawal symptoms such as anxiety and depression when they reduce or stop drinking, which will affect their abstinence compliance. Negative emotional states will affect and induce craving (59). Negative emotions and subjective stress levels are clearly predictors of relapse after AD treatment (60). In our study, the PHQ-9 assessed patients’ severity of depressive symptoms. Our study found that the PHQ-9 scale score reduction rate significantly affected treatment over time, as shown by the interaction effect. At week 2 of follow-up, the right rTMS + C0 group and the left rTMS + C0 group improved depressive symptoms are better. Previous studies have shown that high-frequency rTMS applied to left DLPFC and low-frequency rTMS applied to right DLPFC are an effective treatment for patients with major depression (61, 62). The above theories suggest that rTMS treatment of AD patients may reduce drinking cravings by improving depression, thereby reducing relapse to drinking.

Our study showed significant reductions in GAD, MoCA, OCDS, PSQI scales score reduction rate, improvements in anxiety, cognition, drinking cravings, and sleep in both the sham rTMS + C0 group, sham rTMS + C1 group, right rTMS + C0 group, right rTMS + C1 group, left rTMS + C0 group, and left rTMS + C1 group. There was continuous improvement at 2 weeks, 2 months, 3 months, and 6 months during the follow-up. However, there was no difference in treatment effect between these groups. In addition, we found an interesting finding that the sham rTMS group also improved sleep, anxiety and depression, cognition, drinking desire, and other aspects during 2 weeks, 2 months, 3 months, and 6 months follow-up. Drinking cravings are known to be sensitive to placebo (63), so it was first considered that sham rTMS might have a placebo effect. Second, neuroplasticity may be the most important mechanism in cognitive recovery (64), abstinence alone can restore cognitive impairment and brain abnormalities in some AD patients. Third, AD is seen as a symptom of a dysfunctional family system in which the alcohol dependent individual interacts with other family members. Family members and/or friends play a supportive and motivational role in AD, improve patients’ adherence to therapy, can prevent relapse, and are important in resolving conflicts caused by alcohol abuse (3).

Our research found that smoking was an independent factor influencing alcohol dependent relapse. Many studies support our results. Nearly half of alcohol dependent patients also smoked, and nicotine dependence was associated with a tremendous urge to drink, an increased risk of relapse after treatment, and more alcohol consumption at the time of relapse (65). Alcohol use disorder patients who actively smoke and quit smoking for fewer days before treatment have a significantly higher chance of relapse within 6 months. Implementing smoking cessation could reduce the risk of alcohol use disorder relapse (66). Our finding result may be that both alcohol and nicotine activate the opioid system in reward-related brain regions, leading to adaptive changes in opioid signaling after prolonged exposure. A previous finding suggests that nicotine can increase drinking activity by modulating μ receptor activity in the ventral tegmental area (67). Our study also showed that alcohol consumption was a risk factor for drinking again. A recent domestic survey showed that the daily alcohol consumption of patients in the relapse group was significantly higher than that of patients in the non-relapse group before withdrawal treatment and that high daily alcohol consumption was an independent risk factor for alcohol dependence on relapse (68). The reason may be that everyday heavy drinking increases the patient’s tolerance to alcohol and damages multiple body organs and systems. They were causing severe physical and psychological damage to the patient, leading to more pronounced withdrawal effects and increased psychological addiction, making the patient more susceptible to alcohol-related stimuli and relapse into drinking after withdrawal (69).

## Conclusion

This study is the first to suggest that the combination of rTMS and CBT may be a potentially effective treatment for reducing relapse. Future research should focus on refining phenotypes to achieve personalized treatment approaches: Alcohol use disorders are complex and multifaceted disorders, and personalized treatment approaches may be the most effective to address this complexity. Given the role of individual differences in neuroregulatory effects and the high degree of heterogeneity in the AD population, the identification of phenotypes (including impaired cognitive function, craving, depressive and anxious mood, alcohol consumption, number of relapses, etc.) and individualized treatment options may be critical in the development of treatment for AD. It’s clear that the recovery process is not linear. In order to avoid relapse, much attention should be paid to the interplay between the aspects according to the bio-psycho-social model in the treatment for AD, as well as to increasing patients’ motivation to quit drinking. However, the present study has several limitations. Caution must be used in interpreting the current results, as the sample size for each AD group is not large and is an initial observation. Further studies with a larger sample are needed to replicate our results. In addition, given that one of the distinguishing features of CBT is its relative duration of effect, further follow-up can be extended to assess efficacy. Clinicians should also assess the lifestyle and family structure of the alcohol dependent patient and their role in the treatment process. Finally, although self-assessment diaries in reporting alcohol consumption are generally considered valid under certain conditions, self-assessment diaries are unreliable and inaccurate. In the future, we will further investigate sensitive and specific biological indicators of recent alcohol consumption as a secondary outcome measure to complement the self-reports obtained from patients.

## Data availability statement

The original contributions presented in the study are included in the article/Supplementary material, further inquiries can be directed to the corresponding authors.

## Ethics statement

The studies involving human participants were reviewed and approved by the First Affiliated Hospital of Harbin Medical University. The patients/participants provided their written informed consent to participate in this study. Written informed consent was not obtained from the individual(s) for the publication of any potentially identifiable images or data included in this article.

## Author contributions

XH, LL, TZ, HM, XHZ, HXW, YP, and YJ contributed to the study conception and design. XH performed the statistical analysis and wrote the manuscript. All authors collected the data, commented on previous versions of the manuscript, and read and approved the final manuscript.

## Abbreviations

AD: alcohol dependence
rTMS: repetitive transcranial magnetic stimulation
CBT: cognitive behavioral therapy
DLPFC: dorsolateral prefrontal cortex
ADS: alcohol dependence scale
OCDS: obsessive compulsive drinking scale
VAS: visual analogue scale
MoCA: montreal cognitive assessment
PSQI: Pittsburgh sleep quality index
GAD-7: generalized anxiety disorder-7
PHQ-9: patient health questionnaire-9.
