# Supplementary material for: Repetitive transcranial magnetic stimulation combined with cognitive behavioral therapy treatment in alcohol-dependent patients: A randomized, double-blind sham-controlled multicenter clinical trial
Source: Front Psychiatry. 2022 Oct 4;13:935491. doi: 10.3389/fpsyt.2022.935491 (PMC9590282; doi:10.3389/fpsyt.2022.935491)
Supplement: Supplementary file 1 [file Data_Sheet_1.PDF]

13. Figure legends

Figure 1S. Consolidated Standards of Reporting Trials (CONSORT) diagram showing the flow of participants through each stage of the trial.

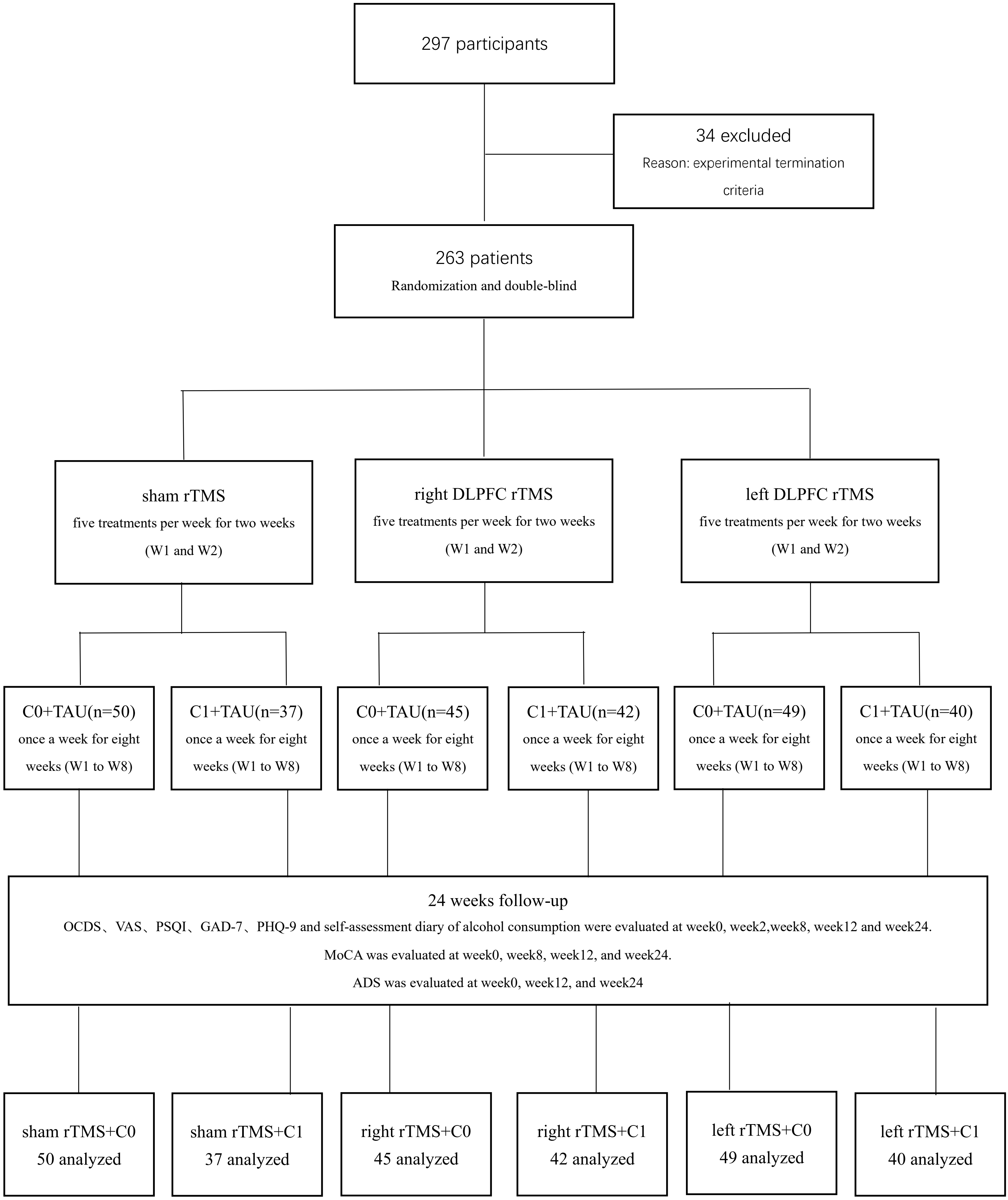

rTMS, repetitive transcranial magnetic stimulation; DLPFC, dorsolateral prefrontal cortex; C0, cognitive behavioral therapy without a fixed plan; C1, cognitive behavioral therapy with a fixed schedule; TAU, treatment as usual; OCDS, obsessive compulsive drinking scale; VAS, visual analogue scale; PSQI, Pittsburgh Sleep Quality Index; GAD-7, Generalized anxiexy disorder-7; PHQ-9, Patient Health Questionnaire-9 items; MoCA, montreal Cognitive Assessment; ADS, alcohol dependence scale

Figure 2S. Kaplan-Meier curves based on relapse rate between six groups in patients with alcohol dependence during follow-up of up to 24 weeks (P = 0.025).

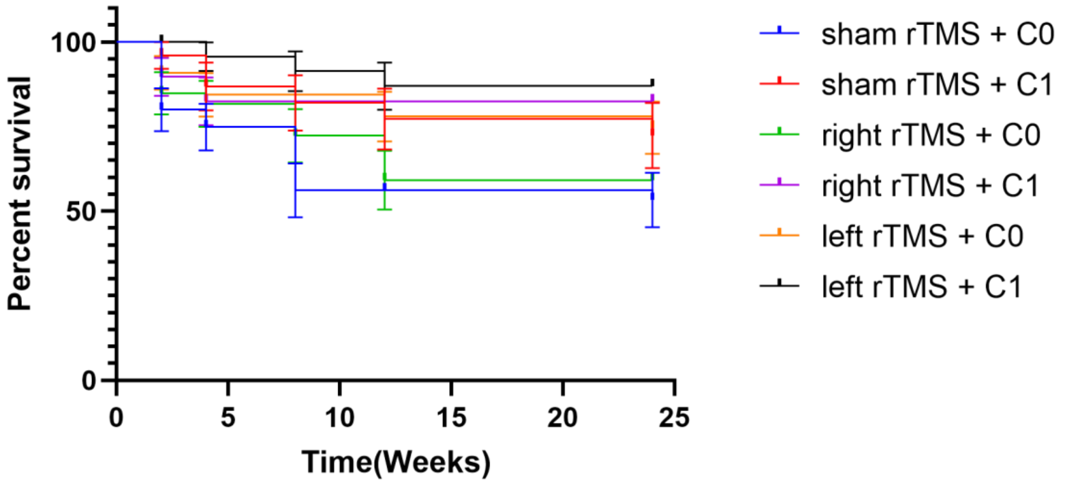

rTMS, repetitive transcranial magnetic stimulation; C0, cognitive behavioral therapy without a fixed plan; C1, cognitive behavioral therapy with a fixed schedule
